# A climatic suitability indicator to support *Leishmania infantum* surveillance in Europe: a modelling study

**DOI:** 10.1016/j.lanepe.2024.100971

**Published:** 2024-06-27

**Authors:** Bruno M. Carvalho, Carla Maia, Orin Courtenay, Alba Llabrés-Brustenga, Martín Lotto Batista, Giovenale Moirano, Kim R. van Daalen, Jan C. Semenza, Rachel Lowe

**Affiliations:** a Barcelona Supercomputing Center (BSC), Barcelona, Spain; b Global Health and Tropical Medicine, Associate Laboratory in Translation and Innovation Towards Global Health, Instituto de Higiene e Medicina Tropical, Universidade NOVA de Lisboa, Lisboa, Portugal; c The Zeeman Institute and School of Life Sciences, University of Warwick, Coventry, United Kingdom; d British Heart Foundation Cardiovascular Epidemiology Unit, Department of Public Health and Primary Care, University of Cambridge, Cambridge, UK; e Heart and Lung Research Institute, University of Cambridge, Cambridge, UK; f Department of Public Health and Clinical Medicine, Section of Sustainable Health, Umeå University, Umeå, Sweden; g Heidelberg Institute of Global Health, University of Heidelberg, Heidelberg, Germany; h Catalan Institution for Research and Advanced Studies (ICREA), Barcelona, Spain; i Centre on Climate Change & Planetary Health and Centre for Mathematical Modelling of Infectious Diseases, London School of Hygiene & Tropical Medicine, London, UK

**Keywords:** Leishmaniasis, Climate change, Infectious diseases, Machine learning, Indicator

## Abstract

**Background:**

Leishmaniases are neglected diseases transmitted by sand flies. They disproportionately affect vulnerable groups globally. Understanding the relationship between climate and disease transmission allows the development of relevant decision-support tools for public health policy and surveillance. The aim of this modelling study was to develop an indicator that tracks climatic suitability for *Leishmania infantum* transmission in Europe at the subnational level.

**Methods:**

Historical records of sand fly vectors, human leishmaniasis, bioclimatic indicators, and environmental variables were integrated in a machine learning framework (XGBoost) to predict suitability in two past periods (2001–2010 and 2011–2020). We further assessed if predictions were associated with human and animal disease data from selected countries (France, Greece, Italy, Portugal, and Spain).

**Findings:**

An increase in the number of climatically suitable regions for leishmaniasis was detected, especially in southern and eastern countries, coupled with a northward expansion towards central Europe. The final model had excellent predictive ability (AUC = 0.970 [0.947–0.993]), and the suitability predictions were positively associated with human leishmaniasis incidence and canine seroprevalence for *Leishmania*.

**Interpretation:**

This study demonstrates how key epidemiological data can be combined with open-source climatic and environmental information to develop an indicator that effectively tracks spatiotemporal changes in climatic suitability and disease risk. The positive association between the model predictions and human disease incidence demonstrates that this indicator could help target leishmaniasis surveillance to transmission hotspots.

**Funding:**

European Union Horizon Europe Research and Innovation Programme (European Climate-Health Cluster), United Kingdom Research and Innovation.

**Research in context**

**Evidence before this study**

Temperature, rainfall, and humidity impact survival and vectorial capacity of sand flies, which transmit human and animal leishmaniasis. In Europe, where leishmaniasis is endemic, previous modelling studies have shown that climate limits sand fly distributions, which could expand under climate change scenarios. However, lack of standardisation in disease reporting has limited the ability to quantify the impact of climate change on human disease. We ran a search in PubMed on 13 Feb 2024 using the string “(leishmaniasis) AND (climate) AND (model) AND (Europe)”. Only two out of 33 studies used human leishmaniasis data in predictive models, but they were restricted to a single country or region. 54% of studies focused on sand flies, without using human disease data. In 2022 and 2023, the European Centre for Disease Prevention and Control published two reports summarising available data on human and animal leishmaniasis, and the distribution of sand flies in the European Union and neighbouring countries.

**Added value of this study**

This study explicitly combined open-access vector and disease data with climate and environmental factors to track climatic suitability for leishmaniasis caused by *Leishmania infantum* across all European countries of the standard Nomenclature for Territorial Units for Statistics (NUTS). We predicted an increase in the number of climatically suitable regions for the disease in the last two decades, coupled with a northward expansion of suitable regions towards central Europe. The predicted climatic suitability was positively associated with human and canine data from France, Greece, Italy, Portugal, and Spain. This suggests that climatic variables influence the distribution of *L. infantum* in these countries. The indicator provides essential and relevant information for regional and local decision-making by identifying subnational regions at higher risk of leishmaniasis transmission.

**Implications of all the available evidence**

The proposed indicator may be used to assess current and future risk at a subnational level, assisting in the development of well-planned surveillance programs. Given its policy relevance, this indicator was selected for the European report of the Lancet Countdown on Health and Climate Change and features in a European Environment Agency report on climate, water and health. This work emerged from a transdisciplinary collaboration, co-developing a suite of indicators and decision-support tools to build climate resilience against emerging infectious diseases in Europe and beyond (IDAlert project), and to better prepare for current and future impacts of climate and environmental changes on human and animal health using sand fly-borne diseases as a model system (CLIMOS project). These projects are part of the European Climate-Health cluster, an Horizon Europe cooperation aimed at increasing the societal and policy impact of EU-funded research linked to climate, human health and climate policy.

## Introduction

Leishmaniases are climate-sensitive diseases caused by *Leishmania* parasites and transmitted by the bites of infected female sand flies (Diptera: Psychodidae: Phlebotominae). Endemic in 99 countries or territories, and estimated to cause 700,000 to 1 million new human cases per year globally, they are highly underreported and disproportionately affect marginalised communities, being associated with malnutrition, population displacement, poor housing, weak immune systems, and lack of financial resources. Hosts include humans, domestic dogs, lagomorphs, synanthropic and wild rodents.^1^^,^^2^ Depending on the *Leishmania* parasite (∼20 known species), human clinical manifestations may vary between: cutaneous leishmaniasis (CL, causing skin ulcers), mucocutaneous leishmaniasis (MCL, causing skin and mucosal ulcers primarily in the nose and mouth), and visceral leishmaniasis (VL, case fatality rate of >95% if not treated). There is no human vaccine, and chemotherapeutic treatments are often toxic, costly, and with low compliance by patients.^2^

The combined effects of temperature, humidity and rainfall impact sand flies in multiple ways. Air temperature is the most influential factor, which influences sand fly metabolism, development, survival rates, and vectorial capacity.^3^ Sand fly distributions tend to be restricted to warmer areas with temperatures above 15 °C for several months, though optimum temperature and humidity conditions vary substantially between species and regions.^4^ Warmer temperatures are expected to reduce the time required for *Leishmania* parasites to develop into the infective stage inside the vector, thus increasing vectorial capacity.^5^ Adult biting activity is constrained by cold summers and by rain, which can likewise kill immature stages.^6^ High soil moisture is important for egg and larvae survival.^7^

Monitoring the interaction between climate, the environment, and disease is crucial for public health surveillance, resource management, and strategic planning - particularly in the context of climate change. Epidemiological, climatic, and environmental indicators that are spatially and temporally resolved can track climatic suitability for infectious diseases and detect early signals of outbreaks.^8^^,^^9^ Global maps of leishmaniasis based on disease occurrence and expert knowledge have been produced, but they lack information on vectors, due to the scarcity of vector data at the global scale.^10^ We developed a climatic suitability indicator for leishmaniasis based on a nested machine learning approach that explicitly considers the relationship between vectors and climate. We demonstrate its application in Europe, where the transmission of *L. infantum* is endemic, and regional human disease and vector data are available,^11^^,^^12^ and climate change is expected to impact the distribution of vectors and the disease epidemiology.^6^^,^^9^^,^^13^

## Methods

Climatic suitability for leishmaniasis is here defined as the probability of a given area having adequate climatic conditions for the occurrence of human and/or animal leishmaniasis caused by *L. infantum*, and adequate environments for its main vectors. Our model objective is to predict climatic suitability, which is expressed in continuous values from 0 (unsuitable) to 1 (100% suitable) at subnational level, in two decadal periods: 2001–2010 and 2011–2020.

### Study area

We used the smallest regional division (NUTS3) of the current Nomenclature for Territorial Units for Statistics (NUTS) classification to generate the climatic suitability predictions. The NUTS classification is a hierarchical system for dividing up the economic territory of the European Union (EU) and the United Kingdom into standardised administrative divisions of 37 countries. Data was obtained at EUROSTAT.^14^

### Response variable: leishmaniasis occurrence

The main *Leishmania* species causing autochthonous VL and CL in Europe is *L. infantum*, with sporadic local transmission of *Leishmania tropica* in Greece (CL) and *Leishmania donovani sensu stricto* in Cyprus (CL). Notification of human and animal cases is not compulsory in all countries, with common underreporting and imported cases.^15^ Between 2013 and 2020, 4399 human leishmaniasis cases were reported in 17 European countries, including 3191 (73%) autochthonous cases (1496 VL and 1695 CL) and 1208 (27%) imported cases (121 VL and 1087 CL).^13^

We used presence or absence of human (VL and CL) and/or animal leishmaniasis caused by *L. infantum* in each NUTS3 region for the period 2009–2020, as reported by the ECDC^11^ (Supplementary Table S1). Records were gathered from an extensive scientific review and questionnaires to public health and veterinary national authorities (Supplementary Fig. S1). Data from non-endemic countries were not used in model calibration to avoid the inclusion of imported cases in regions without local transmission. We considered the current list of endemic countries by the ECDC and the WHO.^11^^,^^16^

### Predictor variables: climate and sand flies

#### Bioclimatic indicators

A set of 19 bioclimatic indicators representing annual averages and seasonal trends of temperature and precipitation was calculated from monthly ERA5-Land climate reanalysis data^17^ for the periods 2011–2020 (for model calibration and prediction), and 2001–2010 (only for retrospective prediction). A comprehensive description of the calculation and selection of bioclimatic indicators is provided in the supplementary file (Supplementary Table S2).

After checking the relative contributions of all 19 bioclimatic indicators to preliminary models and removing those with high collinearity (Pearson’s r > 0.8), nine indicators were selected: annual mean temperature (BIO01), diurnal range of temperature (BIO02), isothermality (BIO03), temperature seasonality (BIO04), maximum temperature of the warmest month (BIO05), minimum temperature of coldest month (BIO06), precipitation of the wettest month (BIO13), precipitation of the driest month (BIO14), precipitation seasonality (BIO15). These were used as predictors in the leishmaniasis and in the vector models.

#### Environmental suitability for sand fly vectors

Approximately 15 *Phlebotomus* sand fly species are proven or suspected vectors of *Leishmania* in the EU and neighbouring countries.^18^ Data from the main sand fly species related to regional transmission of *L. infantum* were included: *Phlebotomus perniciosus*, *Phlebotomus ariasi*, *Phlebotomus perfiliewi*, *Phlebotomus neglectus*, and *Phlebotomus tobbi*. Records by NUTS3 regions, based on historical data and confirmed by local experts, were obtained from the ECDC.^12^ Distribution status categories were reclassified as present (present or introduced) or absent (anticipated or confirmed absent) (Supplementary Fig. S4).

Instead of directly incorporating vector presence/absence in the model as categorical predictors, we estimated their environmental suitability from species-specific models based on the five selected bioclimatic indicators, land cover, and elevation data. The percent coverage of five land cover classes by NUTS3 region was calculated using data from the CORINE Land Cover^17^^,^^19^ project: artificial surfaces, agricultural areas, forest and semi-natural areas, wetlands, and water bodies. Average elevation in metres above sea level by NUTS3 region was calculated from SRTM data, obtained at the WorldClim database.^18^^,^^20^

The model outputs for each vector species (expressing their environmental suitability) were used as predictors in the leishmaniasis models (Supplementary Fig. S7). We opted for this two-step nested modelling approach after prior sensitivity analysis demonstrated its higher predictive power (see Supplementary file), and because these environmental variables are directly related to vector ecology and distribution.^4^^,^^6^^,^^7^^,^^18^

### Model calibration and validation

Models were based on extreme gradient boosted regression (XGBoost), a machine learning algorithm that creates an ensemble of decision trees to form a stronger prediction by iteratively learning from weak classifiers and adding them to a strong classifier (i.e., boosting). XGBoost was recently applied to predict the risk of West Nile Virus outbreaks in Europe,^21^ and global distribution maps of leishmaniasis were produced by the similar boosted regression trees method.^10^ XGBoost is flexible in allowing for non-linearity, both among covariates and between covariates and predictions, and non-random patterns of missing data. It also includes a scale term for weighting unbalanced input datasets, and allows the use of regularisation parameters to prevent overfitting. Models were fitted using the R package xgboost version 1.7.5.1.^22^

Model predictive performance was assessed by the area under the ROC curve (AUC). Alternative methods for defining the model validation datasets were assessed (see Supplementary file), by either randomly removing 20% of the data from the calibration dataset or by excluding the data from different groups of countries of the four European subregions (Southern, Eastern, Western, and Northern). After model sensitivity analysis, the final model input dataset was randomly split into sets for model calibration (80%) and validation (20%).

The model calibration set was applied in 10-fold cross-validation mode for 500 runs to estimate the best parameters for the final models. For each run of the cross-validation, multiple combinations of XGBoost parameters were evaluated (see Supplementary file for details). The selected best parameter values and the full calibration dataset were used to run 1000 replicates of the final models, then further evaluated by the AUC against the independent validation set (Supplementary Fig. S6). We computed 95% confidence intervals for the AUCs by running 2000 bootstrap replicates. After running sensitivity analysis by testing multiple combinations of predictors (see Supplementary file), the model with the best final AUC value was selected to generate climatic suitability predictions for all NUTS3 regions in the two periods (2001–2010 and 2011–2020).

### Model post-processing

Predicted climatic suitability ranges from 0 to 1, where 1 indicates 100% suitable. To quantify the number of suitable and unsuitable NUTS3 regions, predictions were converted to binary (suitable/unsuitable) values by applying the threshold value at which, according to the ROC curves, both sensitivity and specificity were maximised. To visualise changing suitability between the two study periods, maps representing suitable and unsuitable NUTS3 regions were overlaid and subtracted in QGIS version 3.30.1.^23^

The model outputs (i.e., climatic suitability for leishmaniasis) were aggregated across varying levels of socioeconomic conditions, to highlight European regions that have a combination of high predicted climatic suitability and high socioeconomic vulnerability. We used the percentage of people at risk of poverty or social exclusion (AROPE), an indicator from the EU’s statistics on income and living conditions that corresponds to the sum of persons who are (i) at risk of poverty (as indicated by their disposable income); and/or (ii) face severe material and social deprivation (as gauged by their ability to afford a set of predefined material items or social activities); and/or (iii) live in a household with very low work intensity. The data was obtained at EUROSTAT.^14^

### Associations between the climatic suitability indicator and human and animal leishmaniasis

To assess whether predicted climatic suitability for leishmaniasis was associated with observed incidence rates of human VL (HumL) and canine seroprevalence of *Leishmania* infection (CanL), we gathered additional datasets from selected countries.

We obtained human VL case counts at the NUTS3 level recorded by the surveillance systems in France, Greece, Spain, and from Hospital Discharge Records in Italy.^11^^,^^24^ Total case counts were obtained for fixed periods, covering different time windows in each country. Annual population size was extracted from the Eurostat Population and Demography database.^14^ To match the differing time windows in case counts, mean population size in the respective time window was computed for each NUTS3 region.

CanL seroprevalence data was obtained from published cross-sectional studies performed in multiple time periods across Spain^25^ and Portugal.^26^^,^^27^ In these, domestic dog serum samples were tested for the presence of *Leishmania* antibodies by either indirect immunofluorescence antibody test (IFAT, Spain) or the direct agglutination test (DAT, Portugal). The number of positive and tested dogs by NUTS3 regions were extracted from the published reports and matched to the corresponding period of the predicted climate suitability for leishmaniasis.

A series of generalised linear mixed models were built using a Bayesian framework for each selected country and HumL/CanL data. The total number of HumL cases per NUTS3 region was assumed to follow a negative binomial distribution. For the CanL models, we assumed a binomial distribution, with the proportion of positive dogs as outcome. To account for unmeasured variability in the spatial patterns of disease dynamics, a modified Besag-York model^28^ was included as random effects. The posterior marginal distribution of the Rate Ratios (RR) for HumL and of the Odds Ratios (OR) for CanL and their 95% credible intervals (CIs), referred to a 10% increase in the climatic suitability, were computed using Integrated Nested Laplace Approximation with the R package R-INLA version 29.09.09.^29^

### Role of the funding source

The funders of this study had no role in study design; in the collection, analysis, and interpretation of data; in the writing of the report; and in the decision to submit the paper for publication.

## Results

**Fig. 1:**
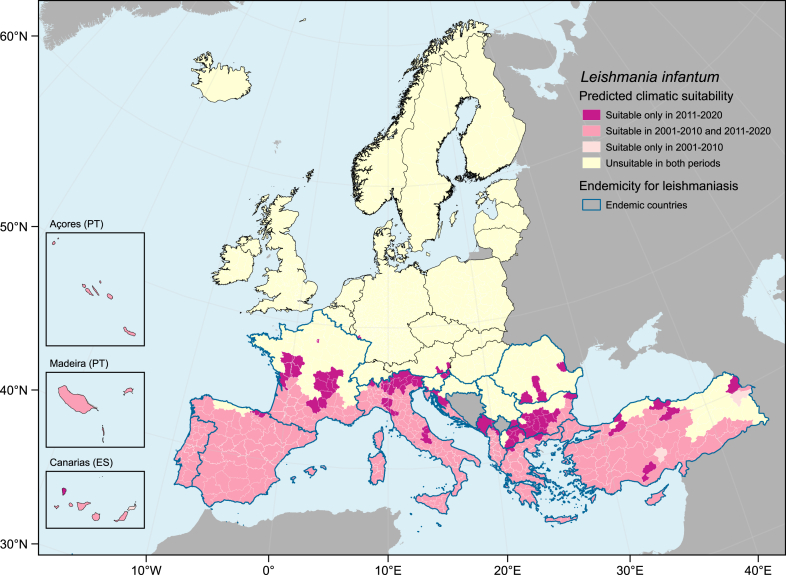
**Predicted change in climatic suitability for *Leishmania infantum* by NUTS3 regions.** Pink shaded areas represent suitable regions in 2001–2010 and 2011–2020. Dark pink areas represent newly suitable regions in 2011–2020. Yellow shaded areas represent unsuitable regions in both periods. Grey shaded areas are countries not included in the models. Countries with blue borders are currently considered endemic for leishmaniasis.

**Fig. 2:**
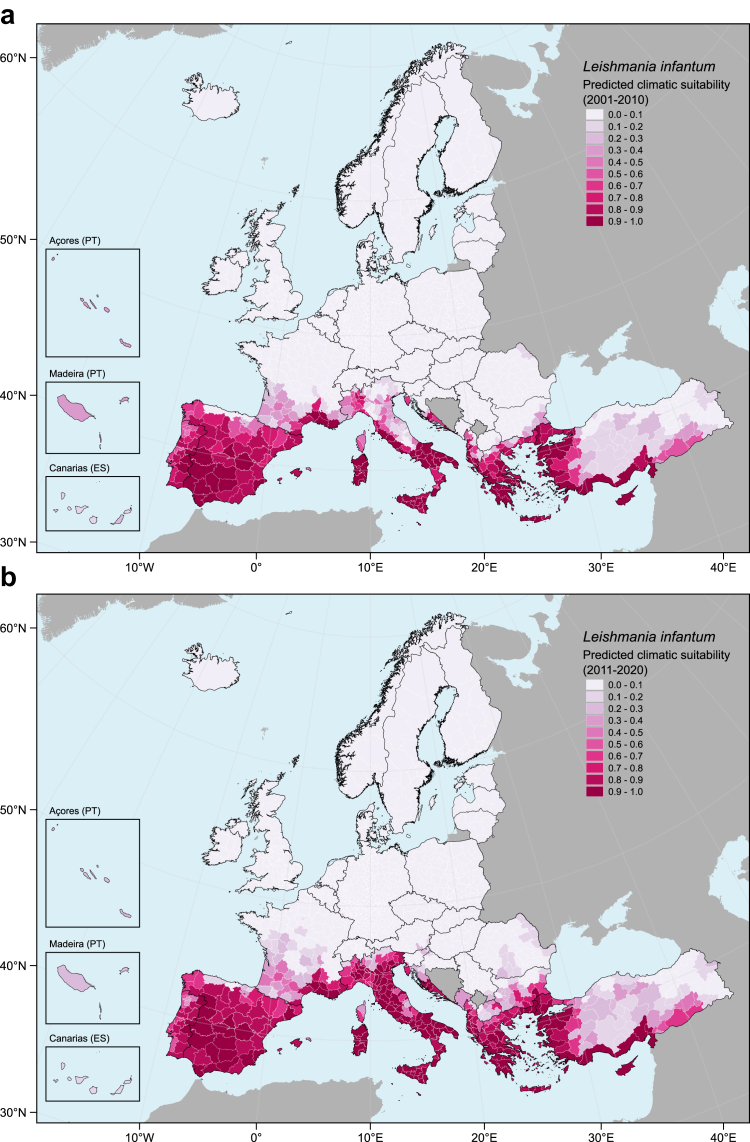
**Predicted climatic suitability for *Leishmania infantum* by NUTS3 regions.** Darker pink colours represent higher climatic suitability by NUTS3 regions, in two decades: a) 2001–2010, and b) 2011–2020. Grey-shaded areas are countries not included in the analysis.

**Fig. 3:**
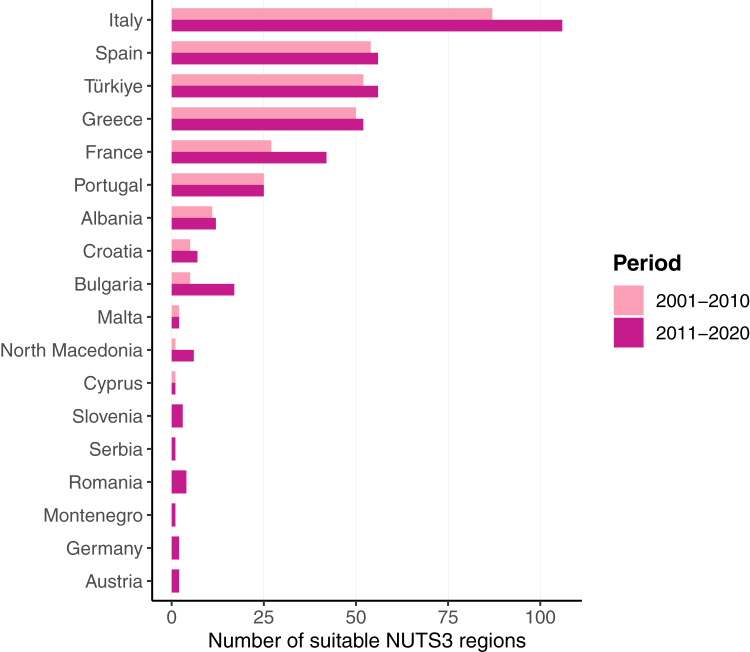
**Number of NUTS3 regions suitable for *Leishmania infantum* transmission by country.** Light pink bars represent the number of suitable regions in 2001–2010; dark pink bars represent the number of suitable regions in 2011–2020.

Climatic suitability for leishmaniasis increased in Europe when comparing 2001–2010 with 2011–2020, more noticeably in southern and eastern Europe (Fig. 1, Fig. 2). The best final model had excellent predictive ability when validated against external data (AUC = 0.970 [0.947–0.993]). The number and spatial distribution of NUTS3 regions predicted to be climatically suitable have changed substantially within each country, between the two decades (Table 1, Fig. 3). Most NUTS3 regions predicted to be suitable in 2011–2020 are located in countries currently endemic for leishmaniasis, except for four NUTS3 regions in Austria and Germany (Table 1, Fig. 1). The number of suitable NUTS3 regions has increased in Italy, Spain, Türkiye, Greece, France, Albania, Croatia, Bulgaria, and North Macedonia (Fig. 3) in 2011–2020 compared to 2001–2010. Slovenia, Serbia, Romania, Montenegro, Germany, and Austria only had predicted suitable NUTS3 regions in 2011–2020 (Fig. 3).

**Table 1:** Total number and percent change of NUTS3 regions suitable for leishmaniasis transmission by country in two periods: 2001–2010 and 2011–2020.

	Total number of NUTS3 regions in country	NUTS3 regions suitable in 2001–2010	NUTS3 regions suitable in 2011–2020	Percent change between periods	Mandatory notification of human or animal cases
Albania^a^	12	11 (91.7%)	12 (100%)	8.3%	Yes
Austria	35	0 (0%)	2 (5.7%)	5.7%	No
Bulgaria^a^	28	5 (17.9%)	17 (60.7%)	42.9%	Yes
Croatia^a^	21	5 (23.8%)	7 (33.3%)	9.5%	Yes
Cyprus^a^	1	1 (100%)	1 (100%)	0.0%	Yes
France^a^	101	27 (28.1%)	42 (43.8%)	14.9%	No
Germany	401	0 (0%)	2 (0.5%)	0.5%	No
Greece^a^	52	50 (96.2%)	52 (100%)	3.8%	Yes
Italy^a^	107	87 (81.3%)	106 (99.1%)	17.8%	Yes
Malta^a^	2	2 (100%)	2 (100%)	0.0%	Yes
Montenegro^a^	1	0 (0%)	1 (100%)	100.0%	Yes
North Macedonia^a^	8	1 (12.5%)	6 (75%)	62.5%	Yes
Portugal^a^	25	25 (100%)	25 (100%)	0.0%	Yes
Romania^a^	42	0 (0%)	4 (9.5%)	9.5%	Yes
Serbia^a^	25	0 (0%)	1 (4%)	4.0%	No
Slovenia^a^	12	0 (0%)	3 (25%)	25.0%	Yes
Spain^a^	59	54 (91.5%)	56 (94.9%)	3.4%	Yes
Türkiye^a^	81	52 (64.2%)	56 (69.1%)	4.9%	Yes

Status of notification of human or animal leishmaniasis.^11^^,^^15^

a Countries currently listed as endemic by the World Health Organization^16^ and the European Centre for Disease Prevention and Control.^11^

**Fig. 4:**
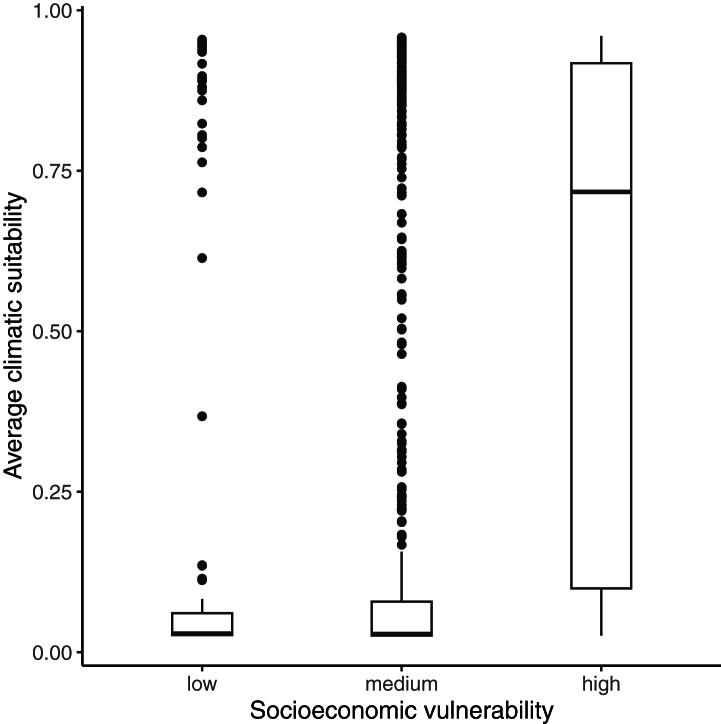
**Climatic suitability for leishmaniasis caused by *Leishmania infantum* in different levels of socioeconomic vulnerability.** The box plots represent average climatic suitability values in NUTS2 regions in 2011–2020.

NUTS2 regions with high socioeconomic vulnerability had higher median values of climatic suitability for leishmaniasis (0.717) when compared with regions with medium and low risk (median climatic suitability 0.029) (Fig. 4). The combination of high socioeconomic vulnerability and high climatic suitability for leishmaniasis was detected in selected regions of Albania, Bulgaria, Greece, Italy, and Spain (Supplementary Fig. S8).

The predicted environmental suitability for the sand fly vectors had good agreement with the known distribution of each species (Supplementary Fig. S5), with higher suitability for *P. perniciosus* (AUC = 0.957 [0.931–0.982]) and *P. ariasi* (AUC = 0.898 [0.0.832–0.963]) in southern and western European regions, and for *P. perfiliewi* (AUC = 0.923 [0.888–0.958]), *P. neglectus* (AUC = 0.949 [0.919–0.978]), and *P. tobbi* (AUC = 0.956 [0.925–0.988]) in eastern and southeastern countries (Supplementary Fig. S8).

**Fig. 5:**
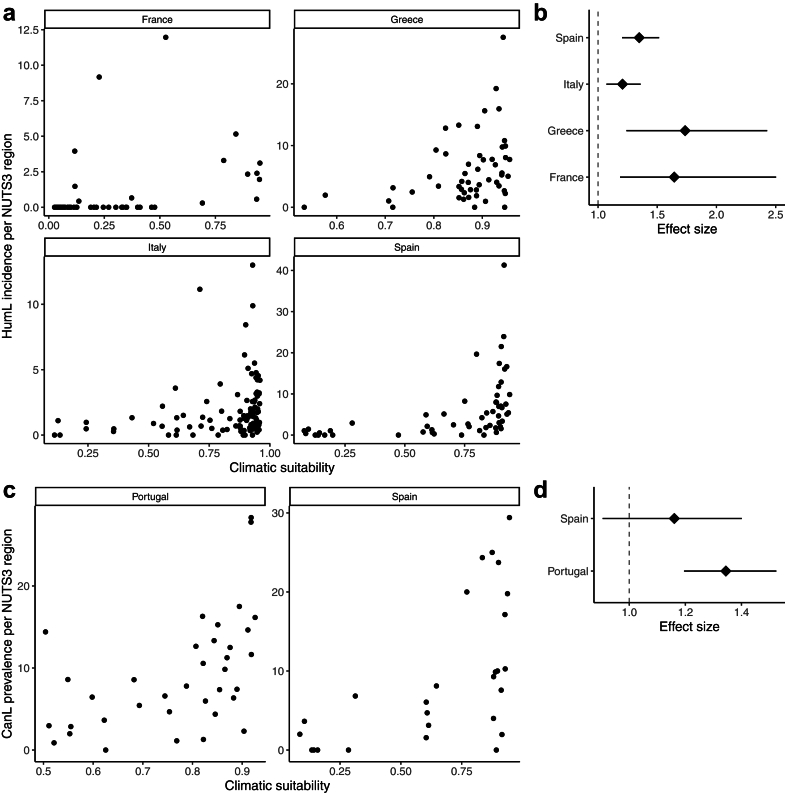
**Associations between predicted climatic suitability for *Leishmania infantum*, human visceral leishmaniasis, and canine seroprevalence of *Leishmania* infection.** a) association between climatic suitability (x-axis) and human visceral leishmaniasis (HumL) incidence per 100,000 inhabitants (y-axis) by NUTS3 region in France, Greece, Italy, and Spain; b) posterior marginal Rate Ratio and 95% credible intervals for a 10% increase in the climatic suitability indicator by country; c) association between climatic suitability (x-axis) and canine seroprevalence of *Leishmania* infection (CanL) (y-axis) by NUTS3 region in Portugal and Spain; d) posterior marginal Odds Ratio and 95% credible intervals for a 10% increase in the climatic suitability indicator by country.

Positive associations were found between HumL incidence and climatic suitability in the selected countries, with the highest effect sizes observed in Greece (RR 1.74 [1.24–2.43]) and France (RR 1.64 [1.19–2.50]), followed by Spain (RR 1.35 [1.20–1.52]) and Italy (RR 1.21 [1.07–1.36]) (Fig. 5). Similarly, CanL was positively associated with predicted climatic suitability in Portugal (OR 1.34 [1.20–1.52]), whereas in Spain the confidence interval was wider (OR 1.16 [0.91–1.40]) (Fig. 5).

## Discussion

Climatic suitability for leishmaniasis in Europe has increased over the past two decades, especially in southern and eastern countries, coupled with a northward expansion towards central Europe. The proposed indicator can be used to monitor changes in climatic suitability as more epidemiological data becomes available. Climate suitability in itself does not necessarily translate into disease transmission probability - climate may be suitable, but leishmaniasis might be absent because of other factors, such as the immunological susceptibility of the population, low vector competence, absence of *Leishmania* parasites, as well as of suitable host species. Our results demonstrate, however, a positive relationship between climatic suitability and both HumL and CanL in selected countries. This is clear evidence that climate is a major determinant of *L. infantum* distribution in Europe, and that the proposed indicator may be used to assess current and future risk at a subnational level, assisting in the development of well-planned surveillance programs.

A growing number of studies have been using machine learning and AI techniques in epidemiological modelling and disease forecasting.^30^ By reducing the assumptions commonly needed by statistical methods regarding data distributions, sampling effort, and multicollinearity, this approach allows for the consideration of non-linear interactions among variables, enhancing the model’s flexibility and predictive accuracy. In this study, a Bayesian hierarchical modelling approach was used to assess whether the predictions produced by a machine learning algorithm agree with epidemiological surveillance data. This framework led to a robust validation of this indicator. In comparison with a previously published global map of leishmaniasis,^10^ which was based on a similar machine learning algorithm, our predictions agree on higher suitability for leishmaniasis in southern Europe.

Decision-making and resource allocation in the public health sector are usually done within standardised administrative divisions, for comparison purposes. One key strength of this indicator is the high spatial resolution of its outputs, making it adaptable to different administrative regions. This is an added value of our approach for public health applications, when compared with previous models that have mostly focused on vector distributions at different scales.^6^^,^^31^ Moreover, the strict spatial stratification of surveillance records poses a challenge for the development of useful decision-making support tools informed by climate processes. In contrast to the adequate spatial resolution of the indicator, its low temporal resolution (decadal) is a limitation constrained by the availability of leishmaniasis epidemiological data. The most recent ECDC report, based on an extensive literature review and questionnaires to health authorities, only includes subnational disease counts for some countries (France, Greece, Italy, Portugal, Spain, and Türkiye), but they are aggregated in different multi-year periods.^11^ A consistent source of yearly disease counts is available by the WHO Global Health Observatory, but only at the national scale.^16^ Nevertheless, the use of climate reanalysis products allowed the comparison of climatic suitability between the two study periods, by calibrating the model with recent leishmaniasis data, and making retrospective predictions up to when climate data is available.

Unprecedented climate change has been observed in Europe in recent decades, with temperatures warming at twice the rate of the global average.^9^ Our indicator predictions show that these climatic conditions are becoming progressively more favourable to local transmission of *L. infantum*. While future projections are not currently available for the disease in Europe, previous studies have predicted a general northward expansion of climatically suitable areas for its vectors, with a likely higher number of competent vector species in Central Europe in the future.^6^^,^^31^^,^^32^ Projecting the climatic suitability for leishmaniasis in Europe into future decades remains a research gap that we aim to incorporate in future updates of the climatic suitability indicator, by adding climate change scenarios and exploring subseasonal to seasonal climate forecasts.^8^^,^^9^

Parts of Europe have historically been endemic for human leishmaniasis. Countries that are moderately or highly endemic include Albania, Bulgaria, Croatia, Cyprus, France, Greece, Italy, Malta, North Macedonia, Portugal, Spain and Türkiye.^13^^,^^15^ The number of climatically suitable regions have increased in the past decade (2011–2020) in most of these countries (Table 1). Bosnia and Herzegovina, Kosovo, Montenegro, Romania and Serbia are countries with low endemicity, where infections in humans and/or animals have been reported in low numbers.^13^^,^^15^ For most of these endemic countries, there are surveillance and control measures in place for human and/or animal leishmaniasis.^15^ In France, where most of its VL autochthonous infections come from the southern region, leishmaniasis is not a notifiable disease.^15^ For Italy, Portugal, and Spain, where VL is of mandatory notification, the number of cases available at the WHO Global Health Observatory was substantially lower than those obtained from hospital discharge databases.^13^ Even in endemic countries, the lack of standardisation in disease reporting limits the execution of European-wide epidemiological studies, and current efforts need to rely on systematic literature reviews and exhaustive contacts to public health agencies.^24^

Despite the presence of sand flies, lack of sustained autochthonous transmission has been reported in Austria, Belgium, Czechia, Germany, Hungary, Luxembourg, Slovakia, Slovenia, and Switzerland.^15^ From these non-endemic countries, our results have predicted climatic suitability for leishmaniasis in four NUTS3 regions of Germany and Austria. *Phlebotomus mascittii* is the predominant sand fly species in central Europe, having historically occupied this region, but its vectorial competence has yet to be confirmed.^32^ In Germany, reports of autochthonous cases exist for humans, dogs, cats, and horses,^33^ while in Austria, leishmaniasis incidence is rising.^34^ Under climate change scenarios, central Europe should progressively become more suitable for sand flies and *L. infantum*.^6^

Regions with both high climatic suitability for leishmaniasis and high social vulnerability are especially at risk of increased leishmaniasis transmission. Climate change can exacerbate food insecurity, and malnutrition is a known risk factor for VL, particularly in children.^35^ Recent evidence shows a reemergence of leishmaniasis in Türkiye, propelled by climate change, urbanisation, and migration.^36^ Inequalities in access to healthcare, social and economic burdens, and stigma associated with leishmaniasis, whilst not as pronounced as in endemic low-income countries, are expected in Europe in lower socioeconomic groups, migrants and refugees, internally displaced, and marginalised populations.^15^^,^^35^ This underscores the urgent need for interventions targeted to the most vulnerable.

The effective control of *Leishmania* hinges upon collaborative approaches that bring together the general public, medical professionals, public health practitioners, and veterinary experts. A crucial aspect is engaging the public through awareness campaigns focused on the disease transmission, emphasising the importance of avoiding vector exposure, especially to socioeconomically vulnerable groups. These should underscore preventive measures, such as using insecticide repellents and installing window screens. For dogs, preventing infection entails the use of insecticide-impregnated collars and spot-on applications, as demonstrated by reductions in incidence through field studies.^37^ Suboptimal adherence to these measures can undermine their success, and integrated strategies are recommended. A successful implementation was observed in Fuenlabrada, Madrid (Spain), where a large-scale outbreak of leishmaniasis was effectively managed using an integrated approach.^38^

Modelling decadal changes in climatic suitability for leishmaniasis at a subnational level offers valuable insights for public health surveillance and preparedness. The observed changes over the past decades suggest that climate change favours the spread of leishmaniasis in Europe. When suitable climatic conditions are combined with socioeconomic inequalities, leishmaniasis risk becomes higher. It is important that countries keep monitoring importation of animal and human cases, especially in those regions where climatic suitability is high and competent vector species are present. Mandatory notification of human and animal cases would improve public health surveillance and in turn foster future epidemiological modelling studies.

## Contributors

Conceptualization: BMC, RL; data curation: BMC, ALB, MLB; formal analysis: BMC, ALB, MLB, GM; funding acquisition: RL, CM; investigation: BMC, ALB, MLB, KRvD, GM; methodology: BMC, MLB; project administration: BMC, RL; resources: RL; software: BMC, ALB, MLB; supervision: RL, CM, OC, JCS; validation: BMC; visualization: BMC, MLB; writing – original draft: BMC, MLB, KRvD, RL; writing – review & editing: all authors.

## Data sharing statement

Original open-access data used in the models are available at their sources, described in the Supplementary file (Supplementary Table S1). The harmonised dataset used in the models and code for reproducing all analysis and results are available at <https://earth.bsc.es/gitlab/ghr/leishmaniasis-europe>.

## Editor note

The Lancet Group takes a neutral position with respect to territorial claims in published maps and institutional affiliations.

## Declaration of interests

The authors declare that they have no conflicts of interest.

## References

[1] González E., Molina R., Iriso A. (2021). Opportunistic feeding behaviour and Leishmania infantum detection in Phlebotomus perniciosus females collected in the human leishmaniasis focus of Madrid, Spain (2012–2018). PLoS Neglected Trop Dis.

[2] WHO (2023). Leishmaniasis fact sheet. https://www.who.int/news-room/fact-sheets/detail/leishmaniasis.

[3] Kasap O.E., Alten B. (2005). Laboratory estimation of degree-day developmental requirements of Phlebotomus papatasi (Diptera: Psychodidae). J Vector Ecol.

[4] Alten B., Maia C., Afonso M.O. (2016). Seasonal dynamics of phlebotomine sand fly species proven vectors of mediterranean leishmaniasis caused by Leishmania infantum. PLoS Neglected Trop Dis.

[5] Hlavacova J., Votypka J., Volf P. (2013). The effect of temperature on *Leishmania* (kinetoplastida: trypanosomatidae) development in sand flies. J Med Entomol.

[6] Koch L.K., Kochmann J., Klimpel S., Cunze S. (2017). Modeling the climatic suitability of leishmaniasis vector species in Europe. Sci Rep.

[7] Moncaz A., Kirstein O., Gebresellassie A. (2014). Characterization of breeding sites of Phlebotomus orientalis – the vector of visceral leishmaniasis in northwestern Ethiopia. Acta Trop.

[8] Rocklöv J., Semenza J.C., Dasgupta S. (2023). Decision-support tools to build climate resilience against emerging infectious diseases in Europe and beyond. Lancet Reg Health Eur.

[9] van Daalen K.R., Tonne C., Semenza J.C. (2024). The 2024 Europe report of the Lancet Countdown on health and climate change: unprecedented warming demands unprecedented action. Lancet Public Health.

[10] Pigott D.M., Bhatt S., Golding N. (2014). Global distribution maps of the leishmaniases. Elife.

[11] ECDC (2022). https://www.ecdc.europa.eu/en/publications-data/surveillance-prevention-control-leishmaniases-European-Union-and-neighbouring-countries.

[12] (2022). ECDC. Phlebotomine sandfly maps. https://www.ecdc.europa.eu/en/disease-vectors/surveillance-and-disease-data/phlebotomine-maps.

[13] Maia C., Conceição C., Pereira A. (2023). The estimated distribution of autochthonous leishmaniasis by Leishmania infantum in Europe in 2005–2020. PLoS Neglected Trop Dis.

[14] (2024). EUROSTAT. Database - Eurostat.

[15] Berriatua E., Maia C., Conceição C. (2021). Leishmaniases in the European union and neighboring countries. Emerg Infect Dis.

[16] WHO (2023). https://www.who.int/data/gho/data/themes/topics/indicator-groups/indicator-group-details/GHO/leishmaniasis.

[17] Copernicus Climate Change Service (2019).

[18] ECDC (2023). The spatial relationship between the presence and absence of Leishmania spp. and leishmaniasis, and phlebotomine sand fly vectors in Europe and neighbouring countries. https://www.ecdc.europa.eu/en/publications-data/spatial-relationship-between-presence-and-absence-leishmania-spp.

[19] (2018). CORINE land cover.

[20] WorldClim (2020). WorldClim. https://www.worldclim.org/.

[21] Farooq Z., Rocklöv J., Wallin J. (2022). Artificial intelligence to predict West Nile virus outbreaks with eco-climatic drivers. Lancet Reg Health Eur.

[22] Chen T., Guestrin C. (2016). Proceedings of the 22nd ACM SIGKDD international conference on knowledge discovery and data mining.

[23] QGIS Development Team (2021). https://www.qgis.org.

[24] Moirano G., Ellena M., Mercogliano P., Richiardi L., Maule M. (2022). Spatio-temporal pattern and meteo-climatic determinants of visceral leishmaniasis in Italy. Trop Med Infect Dis.

[25] Gálvez R., Montoya A., Cruz I. (2020). Latest trends in Leishmania infantum infection in dogs in Spain, Part I: mapped seroprevalence and sand fly distributions. Parasites Vectors.

[26] Cortes S., Vaz Y., Neves R., Maia C., Cardoso L., Campino L. (2012). Risk factors for canine leishmaniasis in an endemic Mediterranean region. Vet Parasitol.

[27] Almeida M., Maia C., Cristóvão J.M. (2022). Seroprevalence and risk factors associated with Leishmania infection in dogs from Portugal. Microorganisms.

[28] Besag J., York J., Mollié A. (1991). Bayesian image restoration, with two applications in spatial statistics. Ann Inst Stat Math.

[29] Rue H., Martino S., Chopin N. (2009). Approximate Bayesian inference for latent Gaussian models by using integrated nested Laplace approximations. J Roy Stat Soc B.

[30] Santangelo O.E., Gentile V., Pizzo S., Giordano D., Cedrone F. (2023). Machine learning and prediction of infectious diseases: a systematic review. Mach Learn Knowledge Extract.

[31] Fischer D., Moeller P., Thomas S.M., Naucke T.J., Beierkuhnlein C. (2011). Combining climatic projections and dispersal ability: a method for estimating the responses of sandfly vector species to climate change. PLoS Neglect Trop Dis.

[32] Kniha E., Dvořák V., Koblmüller S. (2023). Reconstructing the post-glacial spread of the sand fly Phlebotomus mascittii Grassi, 1908 (Diptera: Psychodidae) in Europe. Commun Biol.

[33] Naucke T.J., Menn B., Massberg D., Lorentz S. (2008). Sandflies and leishmaniasis in Germany. Parasitol Res.

[34] Riebenbauer K., Czerny S., Egg M. (2024). The changing epidemiology of human leishmaniasis in the non-endemic country of Austria between 2000 to 2021, including a congenital case. PLoS Neglected Trop Dis.

[35] Hotez P.J., Gurwith M. (2011). Europe’s neglected infections of poverty. Int J Infect Dis.

[36] Tunalı V., Özbilgin A. (2023). Knock, knock, knocking on Europe’s door: threat of leishmaniasis in Europe with a focus on Turkey. Curr Res Parasitol Vect Borne Dis.

[37] Gálvez R., Montoya A., Fontal F., Martínez De Murguía L., Miró G. (2018). Controlling phlebotomine sand flies to prevent canine Leishmania infantum infection: a case of knowing your enemy. Res Vet Sci.

[38] Fuster F., San Martín J.V., Mongue B. (2023).

