# Supplementary material for: A climatic suitability indicator to support Leishmania infantum surveillance in Europe: a modelling study
Source: Lancet Reg Health Eur. 2024 Jun 27;43:100971. doi: 10.1016/j.lanepe.2024.100971 (PMC11261136; doi:10.1016/j.lanepe.2024.100971)
Supplement: Supplementary Figs. S1–S8 and Tables S1–S5 [file mmc1.docx]

# Supplementary Information

# A climatic suitability indicator to support *Leishmania infantum* surveillance in Europe: a modelling study

Bruno M. Carvalho, Carla Maia, Orin Courtenay, Alba Llabrés-Brustenga, Martín Lotto Batista, Giovenale Moirano, Kim R. van Daalen, Jan C. Semenza, Rachel Lowe

**Corresponding authors:** Bruno M. Carvalho [bruno.carvalho@bsc.es], Rachel Lowe [rachel.lowe@bsc.es]. Barcelona Supercomputing Center (BSC), Plaça d’Eusebi Güell 1-3, 08034 Barcelona, Spain.

This supplementary file details about the data pre-processing and modelling methods applied in the development of the climatic suitability for leishmaniasis indicator. A summary of the data sources, original formats, and applied transformations is available at Table S1 below.

**Table S1: Summary of data used in the development of the leishmaniasis climatic suitability indicator.** Data sources, spatial and temporal resolutions are described (with additional modifications between brackets), and at which analysis step the data was used. VL: visceral leishmaniasis.

|  | Source | Spatial resolution | Temporal resolution | Used in |
| --- | --- | --- | --- | --- |
| Human and/or animal leishmaniasis caused by *Leishmania infantum* (presence/absence) | ECDC^1^ | NUTS3 | Historical, 2009-2020 | Leish model calibration |
| Occurrence of sand flies (presence/absence) | ECDC^2^ | NUTS3 | Historical, until 2022 | Vector models calibration |
| Temperature, precipitation | ERA5-Land, Copernicus^3^ | 0.1 x 0.1 degree (averaged by NUTS3) | Monthly, 2001-2020 (averaged for 2001-2010 and 2011-2020) | Bioclimatic indicators |
| Bioclimatic indicators | Calculated from ERA5-Land | NUTS3 | 2001-2010 and 2011-2020 | Leish and vector models calibration, predictions |
| Land cover | CORINE, Copernicus^4^ | 100 x 100 metres (Percent coverage by NUTS3) | 2018 | Vector models calibration |
| Elevation | SRTM, WorldClim^5^ | 2.5 x 2.5 arc-minutes (averaged by NUTS3) | - | Vector models calibration |
| Population at risk of poverty and social exclusion (AROPE) | EUROSTAT^6^ | NUTS2 | Annual, 2021-2022 (averaged and reclassified as low, medium, and high) | Leish model post-processing |
| Human cases of VL in Greece | ECDC^1^ | NUTS3 | Total counts in 2009-2018 | Suitability-disease associations |
| Human cases of VL in Spain | ECDC^1^ | NUTS3 | Total counts in 2000-2018 | Suitability-disease associations |
| Human cases of VL in France | ECDC^1^ | NUTS3 | Total counts in 1999-2012 | Suitability-disease associations |
| Human cases of VL in Italy | Moirano et al. 2022^7^ | NUTS3 | Total counts in 2010-2017 | Suitability-disease associations |
| Population size in Greece, Spain, France, and Italy | EUROSTAT^8^ | NUTS3 | Annual, 2009-2028 | Suitability-disease associations |
| Canine seroprevalence of *Leishmania* infection in Spain | Gálvez et al. 2020^9^ | NUTS3 | 2011-2016 | Suitability-disease associations |
| Canine seroprevalence of *Leishmania* infection in Portugal | Cortes et al 2012^10^, Almeida et al. 2022^11^ | Districts (georeferenced and converted to NUTS3) | 2009, 2021 | Suitability-disease associations |
| European territorial division (NUTS) | EUROSTAT^8^ | NUTS3 | - | Data processing |

## Pre-processing response variable

The main response variable of the climatic suitability model was composed of historical records (presence/absence) of human and/or animal leishmaniasis (VL and CL) caused by *Leishmania infantum* (Figure S1). The data comes from a recent report of the European Centre for Disease Prevention and Control (ECDC)^1^ which summarises the occurrence of leishmaniasis in the Europe an Union and neighbouring countries from an extensive literature review and questionnaires for local health authorities and leishmaniasis experts.

Because this is a longitudinal leishmaniasis dataset based on passive epidemiological surveillance and scientific reports, we assumed that NUTS3 regions with missing data has no records of leishmaniasis transmission (absences). The total sample size used in the models was of 1508 NUTS3 regions, of which 349 presences and 1159 absences.


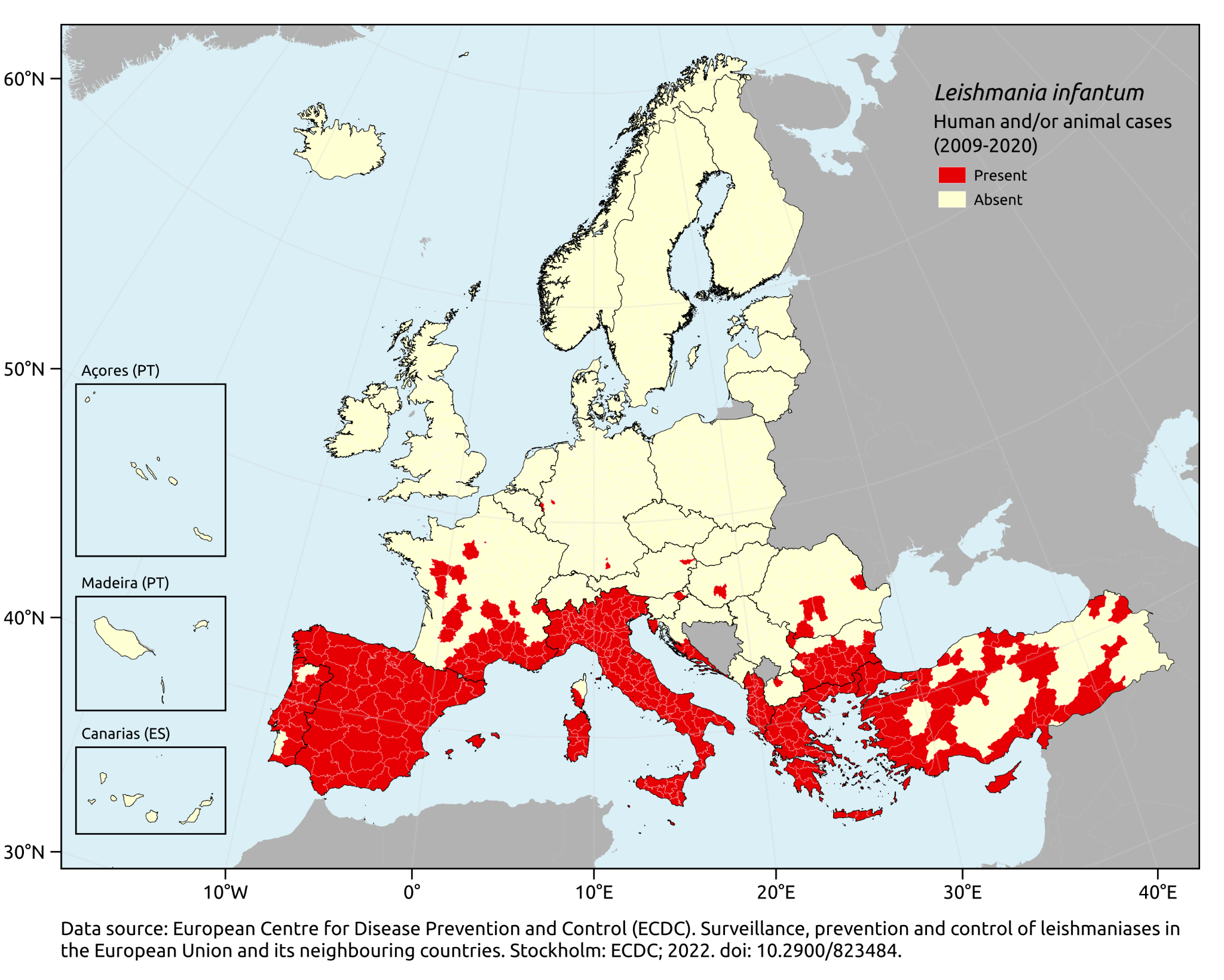


**Figure S1:** Presence/absence of human and/or animal leishmaniasis caused by *Leishmania infantum* in the European Union and the United Kingdom by NUTS 3 region (2009-2020). Data from the European Center for Disease Prevention and Control.^1^

## Pre-processing predictor variables

### Bioclimatic indicators

Temperature and precipitation data from ERA5-Land climate reanalysis were initially obtained at the Copernicus Climate Data Store at 0.1 x 0.1 degree hourly resolution. The hourly values were aggregated to monthly averages for the period of 2001-2020, and then further averaged over two decadal periods (2001-2010, 2011-2020). The original gridded values were spatially aggregated into the NUTS3 regions by zonal averaging. After these transformations, the two sets of decadal monthly averages of temperature and precipitation by NUTS3 regions were used in the calculations of the 19 bioclimatic indicators following their standard formulas (Table S2).

**Table S2**: Definitions and units of the bioclimatic indicators.

|  | **Calculation** | **Units** |
| --- | --- | --- |
| BIO1: Annual Mean Temperature | $Bio1=\frac{\sum_{i=1}^{i=12} {Tavg}_{i}}{12}$ | Degrees Celsius |
| BIO2: Mean Diurnal Range of Temperature  (Mean of the monthly temperature ranges: monthly maximum minus monthly minimum) | $Bio2=\frac{\sum_{i=1}^{i=12} ({Tmax}_{i}-{Tmin}_{i})}{12}$ | Degrees Celsius |
| BIO3: Isothermality  (Quantifies how large the day-to-night temperatures oscillate relative to the summer-to-winter oscillations) | $Bio3=\frac{Bio2}{Bio7}\times100$ | Percent |
| BIO4: Temperature Seasonality  (The amount of temperature variation over a given year based on the standard deviation of monthly temperature averages) | $Bio4=SD\left\{ {Tavg}_{1}, ..., {Tavg}_{12} \right\}$ | Degrees Celsius (x100) |
| BIO5: Max Temperature of Warmest Month | $Bio5=max\left( \left\{ {Tmax}_{1}, ..., {Tmax}_{12} \right\} \right)$ | Degrees Celsius |
| BIO6: Min Temperature of Coldest Month | $Bio6=min\left( \left\{ {Tmin}_{1}, ..., {Tmin}_{12} \right\} \right)$ | Degrees Celsius |
| BIO7: Temperature Annual Range  (A measure of temperature variation over a year) | $Bio7=Bio5-Bio6$ | Degrees Celsius |
| BIO8: Mean Temperature of Wettest Quarter | $Bio8=\frac{\sum_{i=1}^{i=3} {Tavg}_{i}}{3}$  (where *i* represents the three consecutive months of the year with the highest total precipitation) | Degrees Celsius |
| BIO9: Mean Temperature of Driest Quarter | $Bio9=\frac{\sum_{i=1}^{i=3} {Tavg}_{i}}{3}$  (where *i* represents the three consecutive months of the year with the lowest total precipitation) | Degrees Celsius |
| BIO10: Mean Temperature of Warmest Quarter | $Bio10=\frac{\sum_{i=1}^{i=3} {Tavg}_{i}}{3}$  (where *i* represents the three consecutive months of the year with the highest average temperature) | Degrees Celsius |
| BIO11: Mean Temperature of Coldest Quarter | $Bio11=\frac{\sum_{i=1}^{i=3} {Tavg}_{i}}{3}$  (where *i* represents the three consecutive months of the year with the lowest average temperature) | Degrees Celsius |
| BIO12: Annual Precipitation  (Sum of all monthly precipitation values) | $Bio12=\sum_{i=1}^{i=12} {PPT}_{i}$ | Millimetres |
| BIO13: Precipitation of Wettest Month | $Bio13=max\left( \left\{ {PPT}_{1}, ..., {PPT}_{12} \right\} \right)$ | Millimetres |
| BIO14: Precipitation of Driest Month | $Bio14=min\left( \left\{ {PPT}_{1}, ..., {PPT}_{12} \right\} \right)$ | Millimetres |
| BIO15: Precipitation Seasonality  (A measure of the variation in monthly precipitation totals over the course of the year: coefficient of variation) | $Bio15=\frac{SD\left\{ {PPT}_{1}, ..., {PPT}_{12} \right\}}{1+\left( \frac{Bio12}{12} \right)}\times100$ | Dimensionless |
| BIO16: Precipitation of Wettest Quarter | $Bio16=\frac{\sum_{i=1}^{i=3} {PPT}_{i}}{3}$  (where *i* represents the three consecutive months of the year with the highest total precipitation) | Millimetres |
| BIO17: Precipitation of Driest Quarter | $Bio17=\frac{\sum_{i=1}^{i=3} {PPT}_{i}}{3}$  (where *i* represents the three consecutive months of the year with the lowest total precipitation) | Millimetres |
| BIO18: Precipitation of Warmest Quarter | $Bio18=\frac{\sum_{i=1}^{i=3} {PPT}_{i}}{3}$  (where *i* represents the three consecutive months of the year with the highest average temperature) | Millimetres |
| BIO19: Precipitation of Coldest Quarter | $Bio19=\frac{\sum_{i=1}^{i=3} {PPT}_{i}}{3}$  (where *i* represents the three consecutive months of the year with the lowest average temperature) | Millimetres |
| Notation for equations | $i$ = month  ${Tavg}_{i}$ = average temperature for month $i$ (ºC)  ${PPT}_{i}$ = total precipitation for month $i$ (mm)  $SD$ = standard deviation |  |

To assess the collinearity in the bioclimatic dataset, we calculated the pairwise Pearson’s correlation between the 19 indicators (Figure S2). A preliminary model was calibrated by using the full set of leishmaniasis data by NUTS3 and all 19 bioclimatic indicators for 2011-2020 to assess variable contributions to its predictive ability (model ID0 on Table S5). This model had an AUC of 0.966 and the top contributing variables were BIO15, BIO04, and BIO01 (Figure S3). The final selection of bioclimatic indicators was determined by a combination of the most contributing and less correlated ones (Pearson’s r > 0.8): BIO01, BIO02, BIO03, BIO04, BIO05, BIO06, BIO13, BIO14, and BIO15.


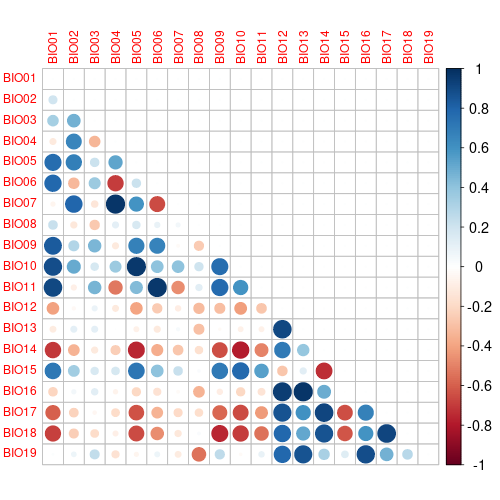


**Figure S2**: Pairwise Pearson correlation matrix of the 19 bioclimatic indicators. The size and colour of the small circles represent the strength and direction of the correlation between the two variables. Bigger circles represent high correlation while smaller circles represent low correlation. Dark-blue represent positive correlation while dark-red represent negative correlation.


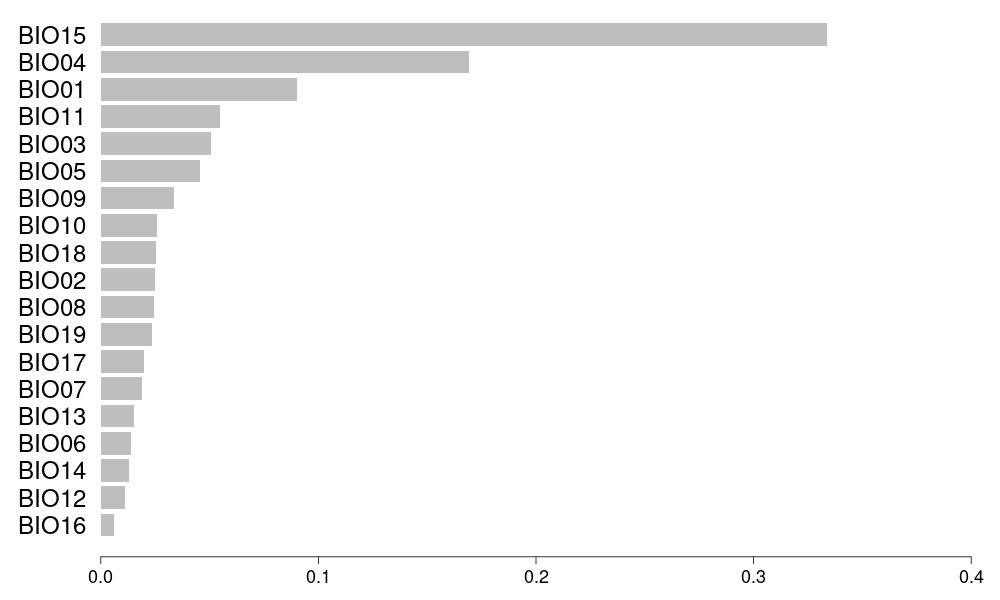


**Figure S3**: Percent contribution of each bioclimatic indicator to the predictive ability of a preliminary model (model ID 0 on Table S5).

### Environmental suitability for sand fly vectors

Historical records (presence/absence) of the five main vector species of *L. infantum* in Europe (*Phlebotomus perniciosus*, *P. ariasi*, *P. perfiliewi*, *P. neglectus*, and *P. tobbi*) by NUTS3 regions were obtained from the ECDC’s VectorNet project website, March 2022 version.^2^ The original distribution status categories (present, introduced, anticipated absent, or confirmed absent) were reclassified as presence (present or introduced) or absence (anticipated or confirmed absent) (Figure S4). For each species, we calibrated a suitability model by adding as predictors: the five selected bioclimatic indicators (BIO01, BIO02, BIO03, BIO04, BIO05, BIO06, BIO13, BIO14, BIO15), percent coverage of five land cover classes, and elevation.

To represent land cover, we obtained data from the CORINE Land Cover project,^4^ a pan-European land cover and land use inventory with 44 thematic classes based on multiple remote sensing products (Sentinel-2 and Landsat-8). We used the 2018 version of the data, reclassified into its five higher order classes (artificial surfaces, agricultural areas, forest and semi-natural areas, wetlands, and water bodies). The percent coverage of each of the five classes in the NUTS3 regions was calculated by zonal statistics using the R package terra version 1.7-71.

Elevation data in meters above sea level was obtained at the WorldClim^5^ database, which in turn was generated from the Shuttle Radar Topography Mission (SRTM). The average elevation values by NUTS3 were calculated from the original grid resolution of 2.5 arc-minutes by zonal statistics using the R package terra version 1.7-71.

As in the leishmaniasis model, we treated missing data in the vector database as absences for calibrating the models. Total sample size for each species was of 1508 NUTS3 regions, with varying number of presences and absences by species (Table S3).

The models for each vector species had good AUC values (Table S3), and their predictions across the European Union were consistent to the known limits of their distribution (Figure S5). These five model outputs, one for each sand fly species, were used as predictors in the leishmaniasis models (Figure S6).

**Table S3**: Sand fly data sample sizes used in the vector models and AUC values with 95% confidence intervals. Data from the VectorNet project (March 2022), European Center for Disease Prevention and Control.^2^

| **Species** | **Number of presence records** | **Number of absence records** | **AUC (95% CI)** |
| --- | --- | --- | --- |
| *Phlebotomus ariasi* | 80 | 1428 | 0.898 (0.832-0.963) |
| *Phlebotomus neglectus* | 136 | 1372 | 0.949 (0.919-0.978) |
| *Phlebotomus perfiliewi* | 161 | 1347 | 0.923 (0.888-0.958) |
| *Phlebotomus perniciosus* | 170 | 1338 | 0.957 (0.931-0.982) |
| *Phlebotomus tobbi* | 96 | 1412 | 0.956 (0.925-0.988) |


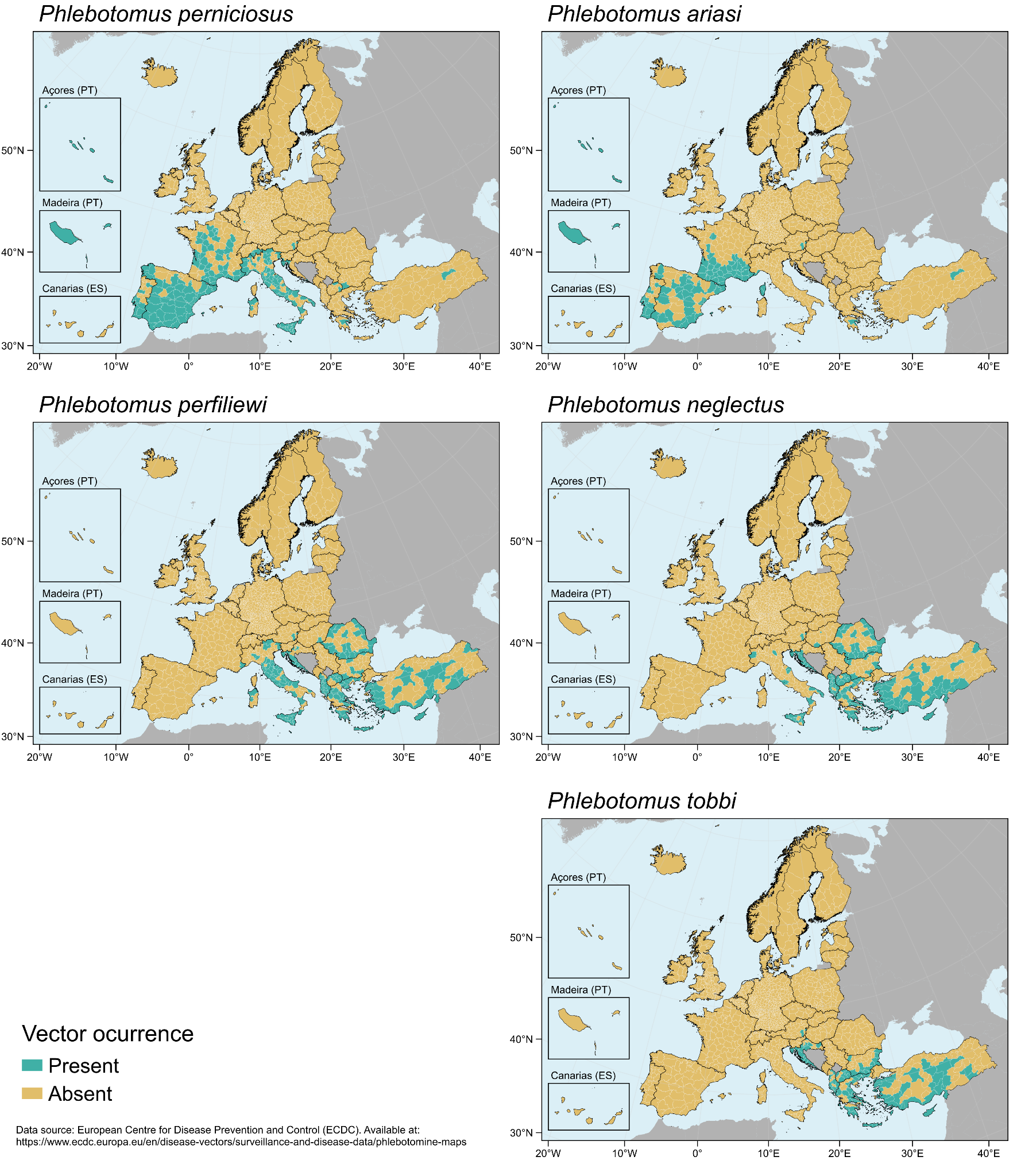


**Figure S4:** Historical presence/absence records of *Phlebotomus perniciosus*, *P. ariasi*, *P. perfiliewi*, *P. neglectus*, and *P. tobbi* in the European Union and the United Kingdom by NUTS3 region. Data from the VectorNet project (March 2022), European Center for Disease Prevention and Control.^2^


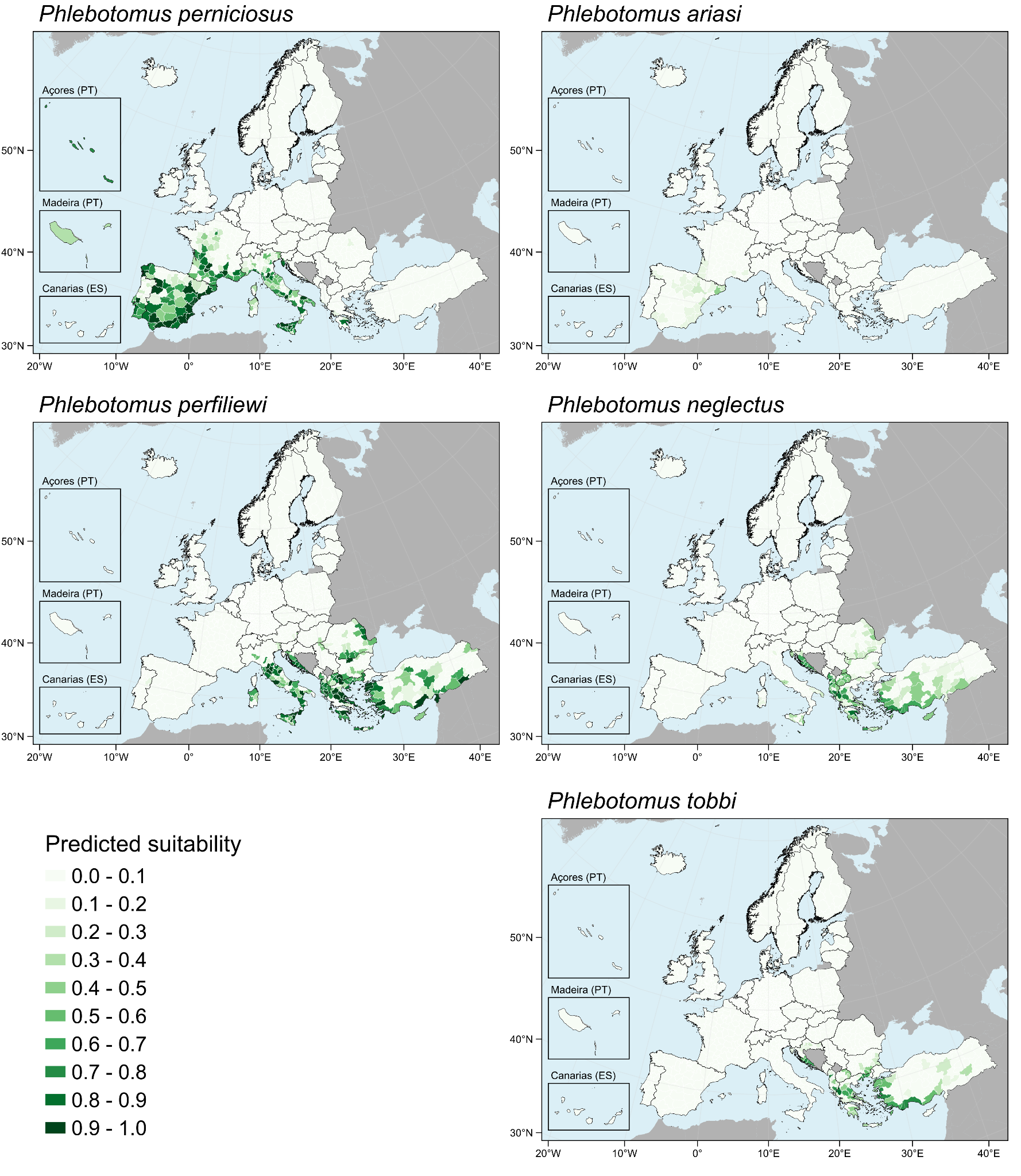


**Figure S5**: Predicted environmental suitability for *Phlebotomus perniciosus*, *P. ariasi*, *P. perfiliewi*, *P. neglectus*, and *P. tobbi* by NUTS3 regions. Darker green colours represent higher environmental suitability.


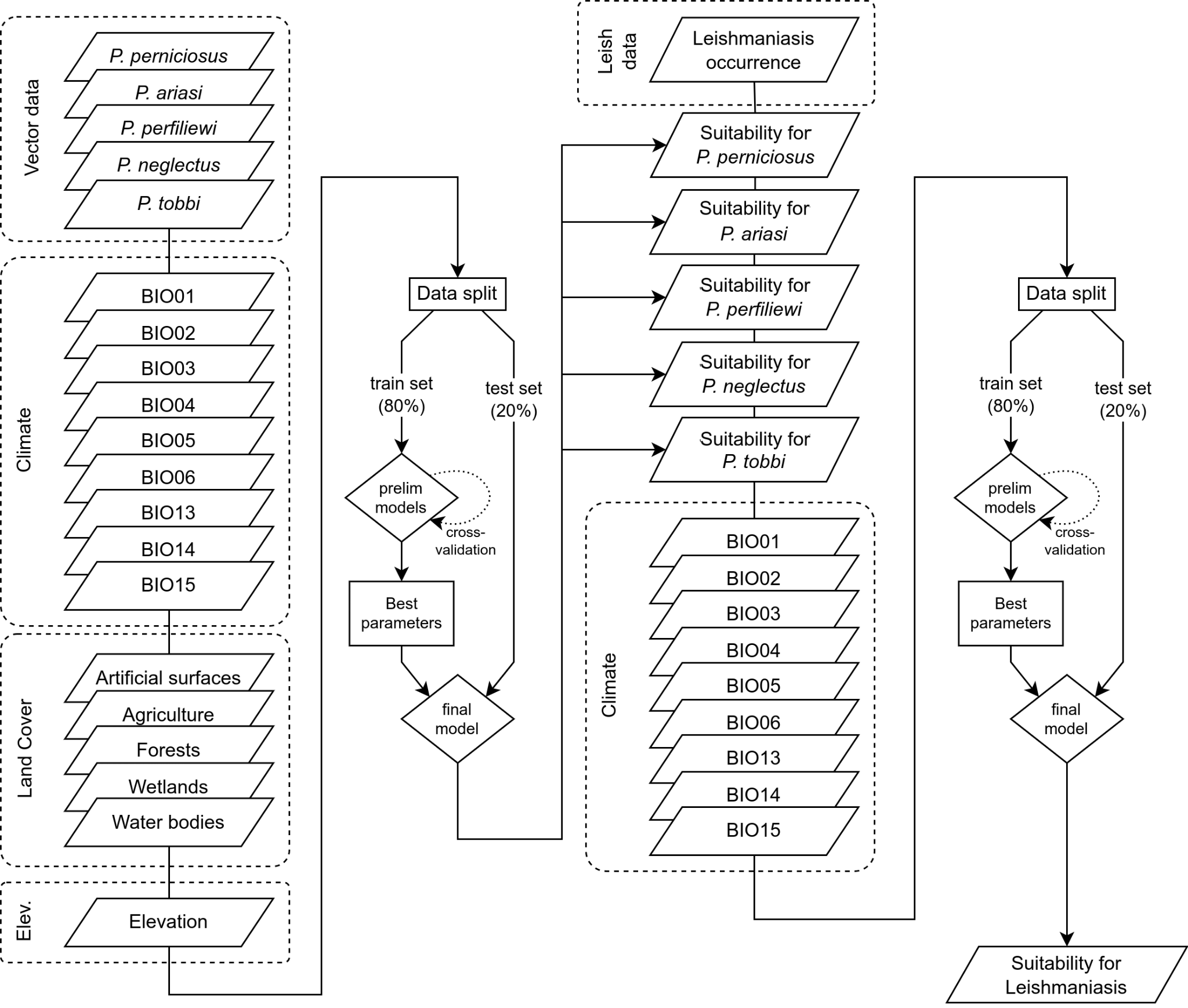


**Figure S6:** Scheme of the nested approach used in model calibration and validation runs, based on the extreme boosted regression (XGBoost) machine learning algorithm.

## Finding the best XGBoost parameters (model calibration)

The XGBoost machine learning algorithm has a set of hyperparameters that control the model training process. Optimal values for these parameters depend on multiple scenarios, and parameter tuning is recommended to improve model predictions and reduce overfitting.^12^ Our approach to estimate the best parameters was based on 10-fold cross-validation runs of the model calibration dataset, where each run had a pre-defined set of parameter values, and their performance was assessed by the AUC. We ran this procedure to find the best values of the following parameters: “eta”, “max_depth”, “max_leaves”, “subsample”, “colsample_bytree”, and “gamma”.

We defined initial sample values of each parameter (Table S4), and then generated a matrix of all possible combinations. For every row of this matrix, 500 model replicates were run in 10-fold cross-validation mode, with an early stopping argument of 10 rounds. The parameter values of the model replicate with the highest AUC value were then used in the final models, which were further validated against out-of-sample data.

**Table S4**: Initial XGBoost parameter values used in model calibration.

| **Parameter** | **Initial values** |
| --- | --- |
| *eta* | 0.001, 0.01, 0.05, 0.1, 0.2, 0.3, 0.4, 0.5 |
| *max_depth* | 15, 17, 19, 21, 23, 25, 27, 29 |
| *max_leaves* | 63, 127, 255, 511, 1023, 2047, 4095 |
| *subsample* | 0.5, 0.6, 0.7, 0.8, 0.9, 1.0 |
| *colsample_bytree* | 0.5, 0.6, 0.7, 0.8, 0.9, 1.0 |
| *gamma* | 0, 1, 2, 3, 4, 5, 6, 7, 8, 9, 10, 11, 12, 13, 14, 15, 16, 17, 18, 19, 20 |

## Model sensitivity analysis

To define the model design with the best predictive ability, we ran candidate models with varying sets of validation data and predictors (Table S5). A preliminary model was run to assess the relative contributions of all the 19 bioclimatic indicators to the model gain, which was validated against 20% randomly excluded data points (Table S5, model ID 0). This same data split method was applied in a set of model runs aimed at finding the best parameter values and set of predictors (Table S5, model IDs 1-5). The multiple combinations of the predictors also included two different methods of incorporating information from sand fly vectors in the models: either directly using the species occurrence data (presence/absence) or their environmental suitability, as predicted by previous models.

By analysing the AUC values of model IDs 1-5, we concluded that the model which includes only vector presence/absence data performed poorly (AUC 0.664), while the ones that included the bioclimatic indicators performed consistently better (AUC ranges 0.968 to 0.970).

Four different validation datasets were additionally assessed, by leaving out of the model calibration data from four random countries, one from each European subregion (Southern, Western, Eastern, and Northern) (Table S5 model IDs 6-9). These models performed consistently well (AUC ranges from 0.918 to 0.976), demonstrating that their predictions generalise well for different European regions.

The model with the best AUC value, which was selected to generate the final predictions of climatic suitability for leishmaniasis, included the predicted suitability for the vectors and the nine selected bioclimatic indicators (model ID 5, AUC 0.970).

**Table S5**: Model candidates run in the sensitivity analysis, their objectives, predictor sets, validation datasets, and AUC with 95% confidence intervals.

| **ID** | **Objective** | **Predictors** | **Validation data** | **AUC (95% CI)** |
| --- | --- | --- | --- | --- |
| 0 | Select bioclimatic indicators | 19 bioclimatic | 20% of all NUTS3 (random) | 0.966 (0.939-0.993) |
| 1 | Find best parameter values and predictors | Vector presence/absence | 20% of all NUTS3 (random) | 0.664 (0.593-0.735) |
| 2 | Find best parameter values and predictors | Vector suitability | 20% of all NUTS3 (random) | 0.888 (0.837-0.938) |
| 3 | Find best parameter values and predictors | Selected bioclimatic | 20% of all NUTS3 (random) | 0.968 (0.945-0.992) |
| 4 | Find best parameter values and predictors | Vector presence/absence + selected bioclimatic | 20% of all NUTS3 (random) | 0.968 (0.939-0.996) |
| 5 | Find best parameter values and predictors | Vector suitability + selected bioclimatic | 20% of all NUTS3 (random) | 0.970 (0.947-0.993) |
| 6 | Find best validation data set (countries) | Vector suitability + selected bioclimatic | NUTS3 regions from Portugal, Germany, Bulgaria, and Denmark | 0.951 (0.914-0.989) |
| 7 | Find best validation data set (countries) | Vector suitability + selected bioclimatic | NUTS3 regions from Spain, Austria, Hungary, and Sweden | 0.918 (0.866-0.970) |
| 8 | Find best validation data set (countries) | Vector suitability + selected bioclimatic | NUTS3 regions from Italy, Switzerland, Romania, and Norway | 0.966 (0.954-0.998) |
| 9 | Find best validation data set (countries) | Vector suitability + selected bioclimatic | NUTS3 regions from Greece, France, Slovakia, and United Kingdom | 0.950 (0.922-0.979) |

## Associations with socioeconomic vulnerability

We used the percentage of people at risk of poverty or social exclusion (AROPE) rates, obtained at EUROSTAT,^6^ to assess socioeconomic vulnerability across the European Union and how it is related with the predicted climatic suitability for leishmaniasis. The original spatial resolution of the AROPE rates is NUTS2 level, so for compatibility with our model outputs, we computed spatial averages of the predicted climatic suitability by NUTS2 regions.

As there are reporting gaps in the AROPE rates between countries and years, and because the calculation methodology changed in 2021, we averaged the available data for each NUTS2 region in 2021-2022 and classified the rates as “low” (0-15%), “medium” (15-30%), and “high” (>30%) (Figure S7). Assuming that these represent recent socioeconomic conditions in the EU, we compared them to the average climatic suitability for leishmaniasis by NUTS2 region in 2011-2020.

Socioeconomic vulnerability is higher in some parts of countries in Eastern and Southern Europe (Figure S7). In these regions, high climatic suitability for leishmaniasis was detected in parts of Albania, Bulgaria, Greece, Italy, and Spain (Figure S8).

## Associations with human and canine leishmaniasis

To assess the degree of association between the predicted climatic suitability and disease dynamics, this study utilised human visceral leishmaniasis (VL) cases at a NUTS3 level, recorded by surveillance systems in France, Greece, and Spain,^1^ as well as Hospital Discharge Records from Italy.^7^ Total case counts per district i (i=1, …, I) were modelled using a Bayesian hierarchical framework independently for each country and assumed to follow a negative binomial distribution with mean μ and overdispersion parameter κ, where:

$$y_{i}|\mu_{i}\sim NegBin(\mu_{i},\kappa)$$

$$\log\left( \mu_{i} \right)=\log\left( p_{i} \right)+log(\eta_{i})$$

Here, μ is defined as the product of the district population size, *p*, at the start of the corresponding period, included as an offset in the model, and the linear predictor η.

$$\log\left( \eta_{i} \right)=\alpha+\beta_{i}x_{t,i}+v_{i}+u_{i}$$

Where α is the intercept, and *x* represents the suitability indicator per district, with the coefficient β estimated using default non-informative priors. To account for the spatial components in the data, a modified Besag-York-Mollie (BYM) model^13^ was employed. This model combines unstructured random effects, built using *iid* hyperparameters, ν, and structured random effects, *u*, built using an intrinsic conditional autoregressive (iCAR) model. The latter implies that neighbouring districts are more similar than non-neighbouring ones.

Outputs were extracted and reported on an exponential scale as risk ratios (RR). Credible intervals including 1 indicate a null association, while values below or above 1 indicates a significant decreasing or increasing association, respectively.

Period-specific seropositivity for canine leishmaniasis (CL) per district was modelled using a similar Bayesian framework, although it was assumed to arise from a binomial distribution with proportion parameter, σ,

$$y_{i}|\sigma_{i}\sim Bin(\sigma_{i})$$

$$\log\left( \sigma_{i} \right)=\alpha+\beta_{i}x_{i}+v_{i}+u_{i}$$

Where α is the intercept, and *x* represents the suitability indicator for each district, with coefficient β. As in the model for human leishmaniasis, the spatial processes in the data were accounted for with a BYM model. Outputs were extracted and reported on an exponential scale as odds ratios (OR). Credible intervals including 1 indicated a null association, while values below or above 1 indicated a significant decreasing or increasing association, respectively.


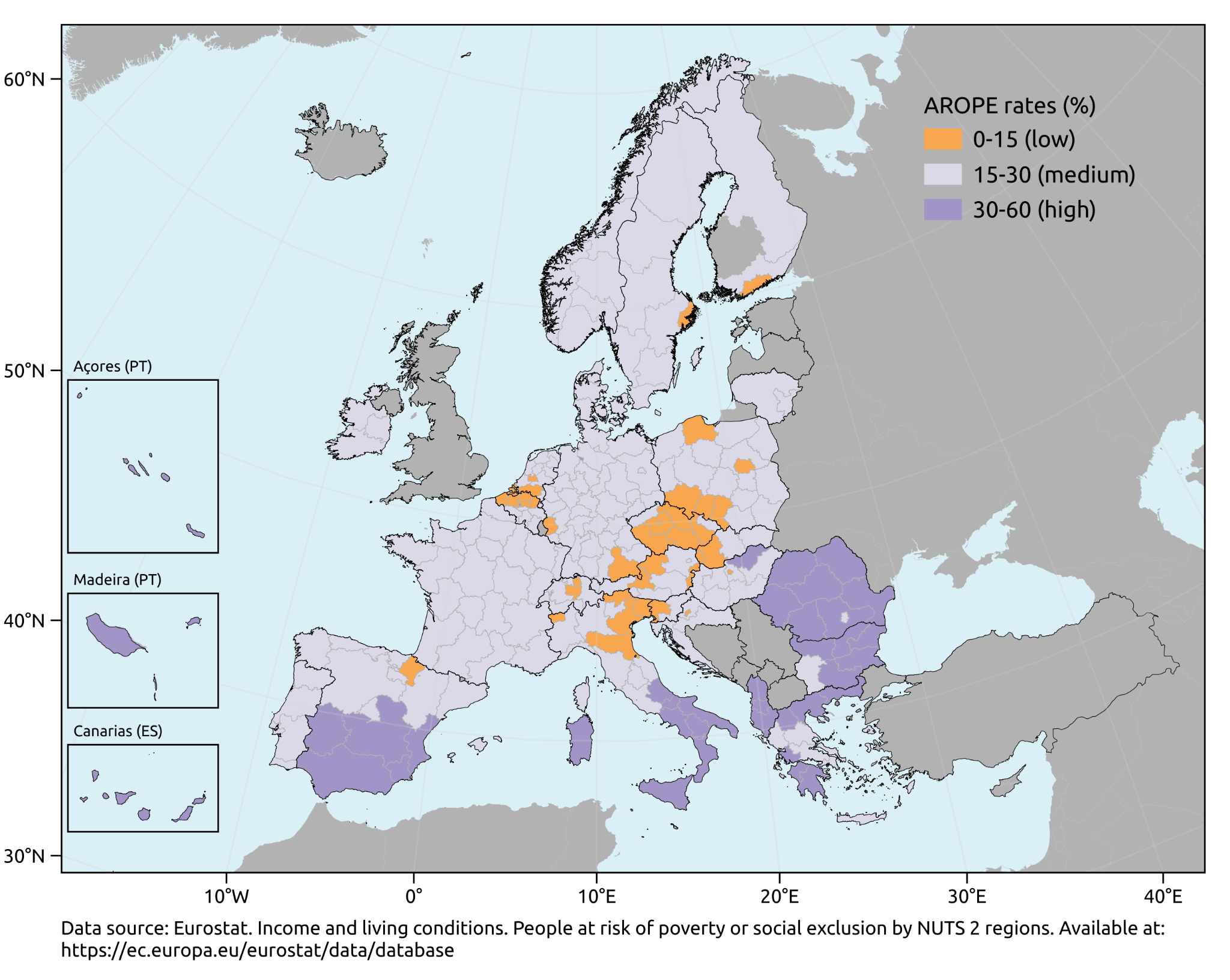


**Figure S7**: Percent population at risk of poverty or social exclusion (AROPE) by NUTS2 regions. Original AROPE rates were reclassified into three categories: “low” (0-15%), “medium” (15-30%), and “high” (>30%). Data from EUROSTAT.^6^


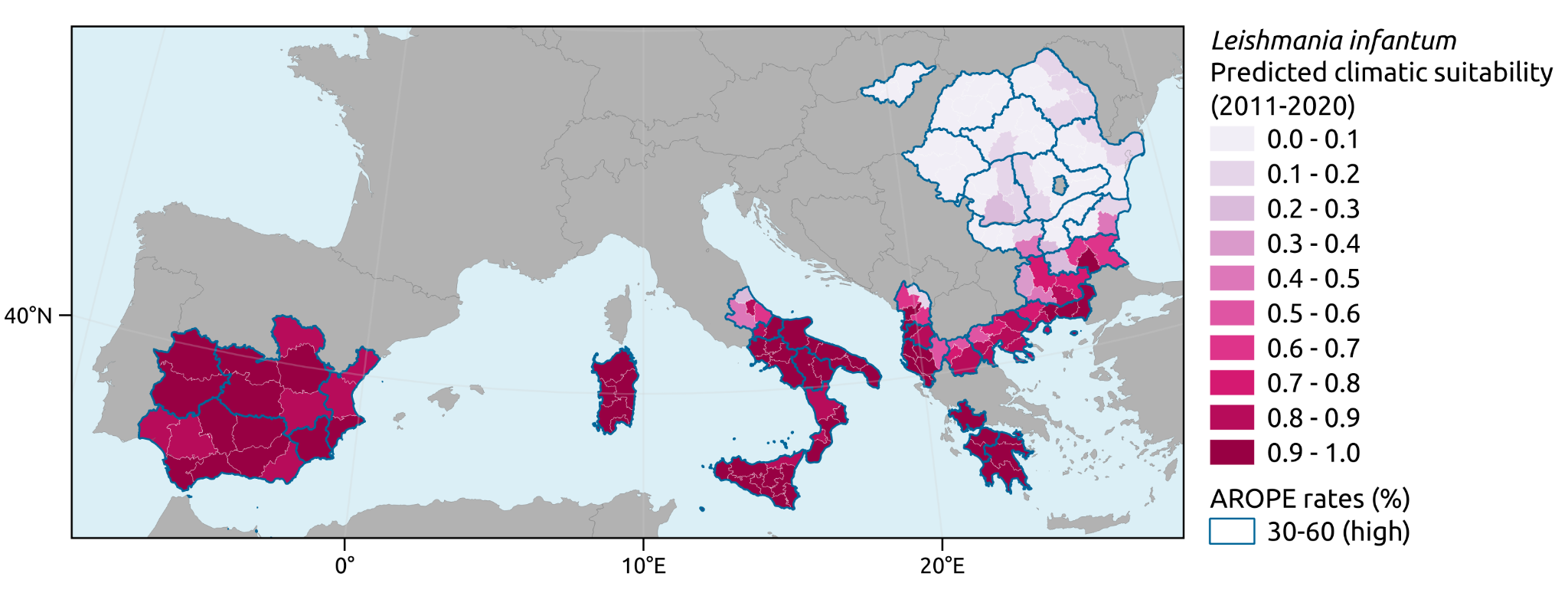


**Figure S8:** Predicted climatic suitability for leishmaniasis caused by *Leishmania infantum* in NUTS2 regions with high socioeconomic vulnerability. Darker pink colours represent higher climatic suitability within the NUTS2 regions with 30-60% population at risk of poverty and social exclusion (AROPE).

## References

1 ECDC. Surveillance, prevention and control of leishmaniases in the European Union and its neighbouring countries. European Centre for Disease Prevention and Control. 2022; published online June 20. https://www.ecdc.europa.eu/en/publications-data/surveillance-prevention-control-leishmaniases-European-Union-and-neighbouring-countries (accessed Dec 2, 2022).

2 ECDC. Phlebotomine sandfly maps. 2022. https://www.ecdc.europa.eu/en/disease-vectors/surveillance-and-disease-data/phlebotomine-maps (accessed Feb 21, 2023).

3 Copernicus Climate Change Service. ERA5-Land monthly averaged data from 1950 to present. 2019. DOI:10.24381/CDS.68D2BB30.

4 Copernicus Land Monitoring Service. CORINE Land Cover. 2018. https://land.copernicus.eu/pan-european/corine-land-cover (accessed June 9, 2023).

5 WorldClim. WorldClim. 2020. https://www.worldclim.org/ (accessed Oct 2, 2020).

6 EUROSTAT. Living conditions in Europe - poverty and social exclusion. 2023. https://ec.europa.eu/eurostat/statistics-explained/index.php?title=Living_conditions_in_Europe_-_poverty_and_social_exclusion (accessed Jan 26, 2024).

7 Moirano G, Ellena M, Mercogliano P, Richiardi L, Maule M. Spatio-Temporal Pattern and Meteo-Climatic Determinants of Visceral Leishmaniasis in Italy. *Tropical Medicine and Infectious Disease* 2022; **7**: 337.

8 EUROSTAT. Database - Eurostat. 2024. https://ec.europa.eu/eurostat/data/database (accessed Jan 12, 2024).

9 Gálvez R, Montoya A, Cruz I, *et al.* Latest trends in Leishmania infantum infection in dogs in Spain, Part I: mapped seroprevalence and sand fly distributions. *Parasites & Vectors* 2020; **13**: 204.

10 Cortes S, Vaz Y, Neves R, Maia C, Cardoso L, Campino L. Risk factors for canine leishmaniasis in an endemic Mediterranean region. *Veterinary Parasitology* 2012; **189**: 189–96.

11 Almeida M, Maia C, Cristóvão JM, *et al.* Seroprevalence and Risk Factors Associated with Leishmania Infection in Dogs from Portugal. *Microorganisms* 2022; **10**: 2262.

12 xgboost developers. Notes on Parameter Tuning — xgboost 2.0.3 documentation. 2022. https://xgboost.readthedocs.io/en/stable/tutorials/param_tuning.html (accessed May 17, 2024).

13 Besag J, York J, Mollié A. Bayesian image restoration, with two applications in spatial statistics. *Ann Inst Stat Math* 1991; **43**: 1–20.
